# 

**Published:** 1896-02

**Authors:** 


### CONTENTS.

### *EDITORIAL.*

PAGE

Arsenicum-album, . . . . . . . . . . . . . . . . . . . . . . . . . . . . . . . . . 61

### *MISCELLANEOUS.*

Allopathic Progress. E. J. Lee, M. D., . . . . . . . . . . . . . . . . . . . . . . . . . . 64

Infinitesimals from a Scientific Standpoint. J. M. Selfridge, M. D., . . . . . . . . . . 68

"The Story of the Liver, by Dr. Andrew Wilson." Arthur Fisher, M. D., . . . . . . . 79

*The Organon* and Materia Medica Club of the Bay Cities of California, . . . . . . . . . 92

*The Organon* and Materia Medica Club of the Bay Cities of California, . . . . . . . . 95

American Institute of Homœopathy, . . . . . . . . . . . . . . . . . . . . . . . . . . 98

Diphtheria and the so-called Anti-toxine. Jos. Fitz Mathew, M. D., . . . . . . . . . . 100

Conditions Affected by Storms. C. Carleton Smith, M. D., . . . . . . . . . . . . . . . 101

### *BOOK NOTICES.*

Diseases of the Respiratory Passages, . . . . . . . . . . . . . . . . . . . . . . . . . 102

Principles of Surgery, . . . . . . . . . . . . . . . . . . . . . . . . . . . . . . . . 104

Travaux d'Electrotherapie Gynécologique, . . . . . . . . . . . . . . . . . . . . . . . 105

Dont's for Consumptives, . . . . . . . . . . . . . . . . . . . . . . . . . . . . . . . 105

Surgical Cleanliness, . . . . . . . . . . . . . . . . . . . . . . . . . . . . . . . . . 106

A Handbook of the Diseases of Children and their Homœopathic Treatment, . . . . 106

Pregnancy, Labor, and the Puerperal State, . . . . . . . . . . . . . . . . . . . . . . 108

